# HMT-Net: Transformer and MLP Hybrid Encoder for Skin Disease Segmentation

**DOI:** 10.3390/s23063067

**Published:** 2023-03-13

**Authors:** Sen Yang, Liejun Wang

**Affiliations:** College of Information Science and Engineering, Xinjiang University, Urumqi 830046, China

**Keywords:** skin lesion segmentation, CTrans module, TokMLP block

## Abstract

At present, convolutional neural networks (CNNs) have been widely applied to the task of skin disease image segmentation due to the fact of their powerful information discrimination abilities and have achieved good results. However, it is difficult for CNNs to capture the connection between long-range contexts when extracting deep semantic features of lesion images, and the resulting semantic gap leads to the problem of segmentation blur in skin lesion image segmentation. In order to solve the above problems, we designed a hybrid encoder network based on transformer and fully connected neural network (MLP) architecture, and we call this approach HMT-Net. In the HMT-Net network, we use the attention mechanism of the CTrans module to learn the global relevance of the feature map to improve the network’s ability to understand the overall foreground information of the lesion. On the other hand, we use the TokMLP module to effectively enhance the network’s ability to learn the boundary features of lesion images. In the TokMLP module, the tokenized MLP axial displacement operation strengthens the connection between pixels to facilitate the extraction of local feature information by our network. In order to verify the superiority of our network in segmentation tasks, we conducted extensive experiments on the proposed HMT-Net network and several newly proposed Transformer and MLP networks on three public datasets (ISIC2018, ISBI2017, and ISBI2016) and obtained the following results. Our method achieves 82.39%, 75.53%, and 83.98% on the Dice index and 89.35%, 84.93%, and 91.33% on the IOU. Compared with the latest skin disease segmentation network, FAC-Net, our method improves the Dice index by 1.99%, 1.68%, and 1.6%, respectively. In addition, the IOU indicators have increased by 0.45%, 2.36%, and 1.13%, respectively. The experimental results show that our designed HMT-Net achieves state-of-the-art performance superior to other segmentation methods.

## 1. Introduction

Dermoscopy is the primary method for increasing skin cancer diagnosis and decreasing skin cancer mortality [1]. Dermoscopy is an imaging technique that eliminates skin surface reflection and enhances deep skin visualization. Due to the different diagnostic equipment for skin diseases and the differences in the quality and size of pictures, different doctors tend to make subjective decisions based on their own experiences in the diagnosis process [2], which often consumes many human and material resources. Because of these problems, dermoscopic image segmentation technology has emerged in recent years.

The currently used standard skin lesion inspection method consists of five steps: imaging, preprocessing, segmentation, feature extraction, and classification [3]. In this process, doctors can only focus on the lesions obtained by segmenting dermoscopic image lesions for a timely diagnosis. Early segmentation methods for skin lesions usually use algorithms based on optimal thresholding [4], region growing [5], and edge detection [6].

Because these traditional methods necessitate human intervention, an increasing number of experts and researchers began to investigate more effective segmentation methods. 

With the success of CNNs in the visual field, people have begun to propose and improve various CNN-based segmentation methods, such as UNet [7], R2U-Net [8], and FAC-Net [9]. However, mainstream CNNs also have their own limitations. The reason is that CNNs do not consider the relationship between remote context information and extracted lesion image features, resulting in a certain semantic gap that leads to the problem of segmentation blur in lesion image segmentation.

In response to the inherent defects of CNNs, many scholars have tried to apply the transformer method in natural language to the field of vision. The core idea of transformer is to use the self-attention mechanism to explore the correlation between global elements. Among them, visual transformer (UTNet [10], MCTrans [11], and UNETR [12]) proved that transformer can achieve excellent results in image classification, image segmentation, image detection, and image tracking.

In addition, many experts try to apply MLP to image segmentation directions. MLP not only has the characteristics of long-term dependence but also has excellent classification ability. Among them, MLP (MLP-Mixer [13], UNeXt [14], and S2-MLP [15]) is also often used in image classification, image segmentation, target detection, and other fields. These methods have also achieved very good results.

Skin lesion tissue is often shown in the form of local blocks, and the shape and size of the lesion area are different. In addition, when we only use convolution operation to extract focal region features and discard useless background information, some important foreground information is often lost or too much background information is retained. To solve the above problems, we designed a hybrid encoder network, HMT-Net. The transformer method we use overcomes CNN’s difficulty capturing remote context connections. Meanwhile, we take advantage of MLP’s focus on local feature learning to make up for the shortcomings of transformer in understanding local feature information. We effectively combined these two methods to achieve better segmentation of skin lesions. The main contributions of HMT-Net are as follows:

We summarize the contributions of this paper as follows:

- We used the CTrans module in a skin disease dataset segmentation task. The transformer in the CTrans module can calculate the correlation between global elements, which enhances the network’s understanding of global information and thereby improves the network’s overall recognition ability for foreground information;
- We used the modules of TokMLP in the network. The TokMLP module performs shift operations on different axial elements in the feature map to improve the correlation between a certain point element and its surrounding elements, which improves the network’s learning ability for lesion edge information;
- By effectively connecting the CTrans module with the TOKMLP block, we designed a new approach based on DLL architecture.

We present existing work in Section 2. We then describe the method and its corresponding analysis in Section 3. The experimental setup, details, and evaluation metrics are presented in Section 4. In Section 5, we present the results of the experiment. Next, we describe the discussion of the network, draw some conclusions, and look to the future in Section 6.

## 2. Related Work

Skin lesion segmentation [16] accurately segments the lesion area manually or by other means to prepare for the doctor’s diagnosis and treatment. Traditional skin lesion segmentation algorithms include threshold-based [4], region-growing [5], edge detection [6], and active contour-based [17] segmentation methods. Although these methods are still widely used in image segmentation tasks, they often set a specific threshold or judge elements based on contours. Similar segmentation algorithms struggle to meet the large number of data points in some lesion areas, with significant changes in image segmentation results. Ben Cohen et al. [18] first explored the use of full convolutional neural networks (FCNs) to achieve the task of liver and tumor segmentation in CT images. A FCN rewrites the classification network as a network for image segmentation by rewriting the fully connected layer as a convolutional layer and using deconvolution to complete the upsampling process. Due to the simple network structure, its segmentation results are not fine enough, and there is still a lot of room for improvement. Yuan et al. [19] completed an end-to-end skin melanoma segmentation method based on 19-layer FCN. The segmentation method based on 19-layer FCN has a deep network layer, and the complexity of the network will increase accordingly.

In recent years, with the continuous development of deep learning in the field of computer vision, the segmentation algorithms of skin lesions have achieved remarkable results. UNet [7] is a landmark work that first uses skip connections to apply the feature information in the encoder to the decoder. Although such a connection method can effectively alleviate the inevitable information loss in the downsampling process, However, different medical image segmentation tasks require different segmentation networks to enhance feature learning for specialized data, so after UNet, many more important medical image segmentation models have emerged, such as UNet++ [20], UNet3+ [21], R2U-Net [8], SA-UNet [22], CE-Net [23], FAC-Net [9], SMU-Net [24], and BA-Net [25]. Although these network architectures can achieve good performance in different tasks, their generalization ability is limited. This is because these networks are all combined using different forms of convolution operations, and convolution operations have obvious limitations; that is, it is difficult to learn the correlation between global elements. As a result of the inherent flaws of the convolution operation, many scholars have attempted to propose alternative methods to solve this problem. 

Recently, many transformer-based networks have been proposed and used for medical image segmentation. The transformer’s overall structure is typically an encoder and decoder structure. Transformer can effectively establish long-distance dependencies through the self-attention mechanism, and the model’s learning ability can be strengthened through multiple heads. The transformer obtains the initial embeddings obtained by the image through the convolution block or directly operates on the pixels and embeds them in the transformer, and this operation does not always need to maintain the original structure of the feature map so that it has a good modality fusion ability. Transformer can perform a variety of tasks by using the attention mechanism. Therefore, it has a wide range of applications in the field of medical image segmentation, such as TransUNet [26], UCTransNet [27], and Swin-Unet [28]. Although these methods can better learn global foreground information in medical image segmentation tasks, their weakness is that it is easy to ignore the learning of local feature information. How we effectively model the overall understanding of foreground information, and the acquisition of local feature information is a problem we need to consider.

In addition, networks based on the MLP architecture have also been proposed and used in the field of image segmentation. MLP has structures such as an input layer, a hidden layer, and an output layer. In image segmentation work, methods based on MLP architecture are often widely used. For example, UNeXt [14], AS-MLP [29], and CycleMLP [30] all use different axial shift operations to interact with spatial information flows in different directions to strengthen the connection between pixels. It can effectively make up for transformer’s weakness in ignoring the understanding of local feature information.

Due to the presence of occlusions, such as hair and air bubbles, in the skin disease images, the shape is different, the boundary is blurred, and the internal and external layer characteristics of the lesion are different. Moreover, when CNNs extracts lesion feature information, it loses some important foreground information when discarding useless background information, which leads to the problem of blurred lesion image segmentation. Aiming at the problems existing in the skin disease data and the CNNs method, we designed a hybrid encoder network, HMT-Net, using the CTrans module to enhance the network’s learning of global elements and improve the network’s ability to understand the global feature information of lesions. Using the TokMLP module can promote the correlation between adjacent elements and make up for the weakness of the transformer ignoring local information through axial shift operations in different directions, thereby improving the network’s ability to learn feature information about the lesion edge.

## 3. Method

This chapter begins with a brief introduction to the overall framework of HMT-Net. Then, the composition, structure, and implementation details of the TokMLP block are introduced. Finally, we introduce the CTrans module in detail.

### 3.1. The Overall Structure of the HMT Network

**Figure 1:**
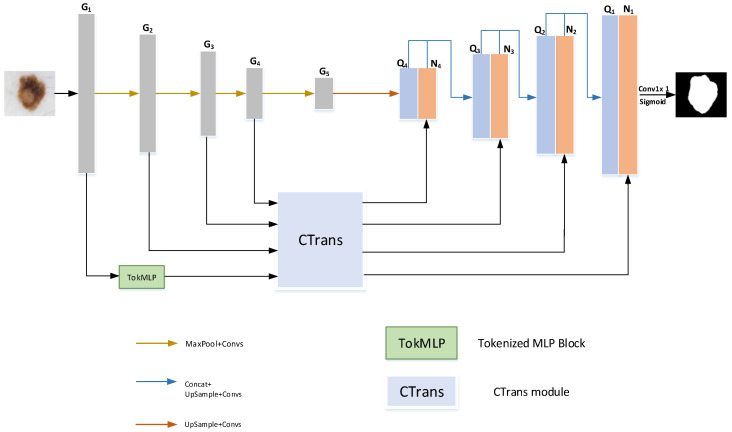
HMT network architecture.

The HMT-Net skin lesion segmentation network is depicted in Figure 1. In other words, we tried various connection methods for the TokMLP block and the CTrans module continuously and finally connected them effectively. We successfully applied it to skip connections. First, the CTrans module we use can effectively calculate the interrelationship of global feature information, which enhances the network’s ability to learn global information. It can effectively improve the overall ability to understand the foreground information of skin damage images. Secondly, we have effectively connected the TokMLP block with the CTrans module (as shown in Figure 1), which can enhance the network’s understanding of local feature information by strengthening the connection between adjacent element points. It can effectively enhance the network’s ability to identify lesion boundaries. The characteristic of this network is that the idea of combining the CTrans module, and the TokMLP block is adopted in the encoder stage. We further combine these two ideas to achieve effective and accurate segmentation of skin lesion feature maps.

### 3.2. TokMLP Block

**Figure 2:**
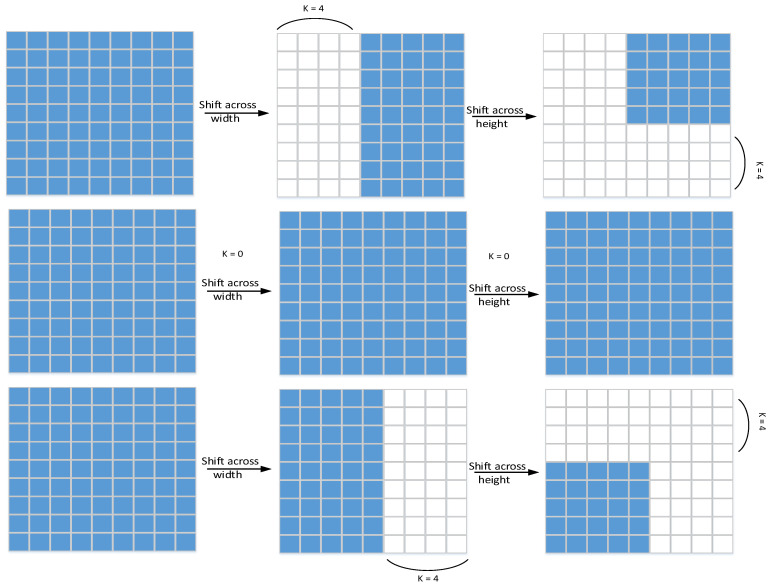
Shift operation schematic diagram. Features move in order in height and width. White is the padding after the move, and blue is the position of the feature block.

We first shift the convolutional features in an orderly manner along the channel axis. Since this operation can enhance MLP’s attention to certain local feature information, the MLP block produces locality. We first move the feature map horizontally in the width and height axes, respectively (the shift operation principle is shown in Figure 2), and its principle is very similar to axial attention [31]. First, the feature map is divided into m partitions, and then they are sequentially moved by k positions according to the specified different axes.

**Figure 3:**
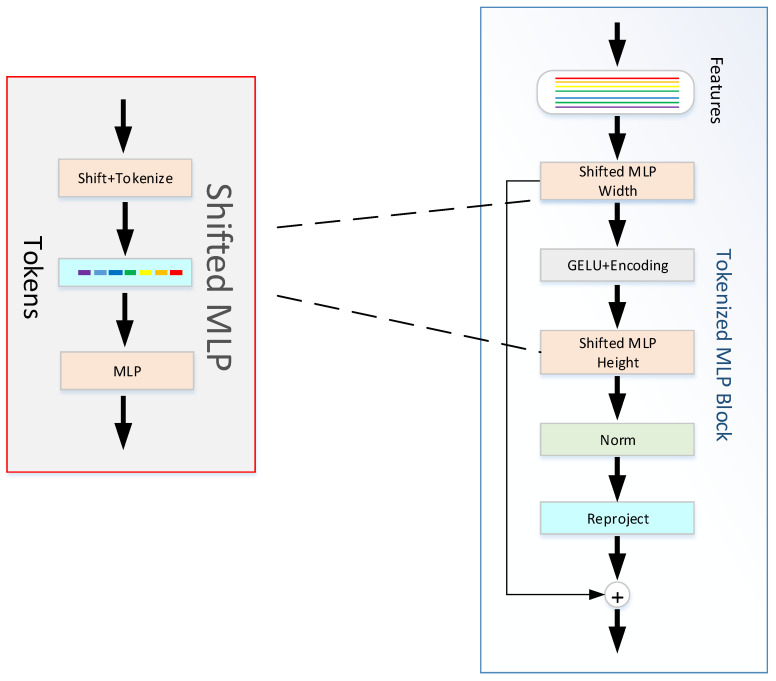
TokMLP block.

In the TokMLP block (Figure 3), we first map the shifted feature information into tokens. Two MLP modules make up the TokMLP block we use (as shown in Figure 3). These tokens are first passed by us to the shifted MLP (span width). Then, this feature information needs to go through the depth direction convolution layer (DWConv) operation. It should be noted that the operation of the GELU [31] activation layer is included in the DWConv operation. GELU is a common activation function, the full name of which is “Gaussian error linear unit”. This activation function has random regular operation. Even state-of-the-art architectures, such as ViT [32] and BERT [33], achieve good results due to the use of GELU. Our approach of using GELU instead of RELU is a smoother alternative with better performance. Next, we need to pass the feature information into another moving MLP (across height). Finally, we feed the layer normalized (LN) features into the next module. LN is a normalization operation on a single neuron in an intermediate layer, and we use LN because in the TokMLP block, layer normalization along tokens makes more sense than batch normalization (BN). The calculation process in the TokMLP block is as follows:

(1) $$X_{shift\;}=Shift_{K}\left(X\right);T_{K}=Tokenize\left(X_{shift}\right)$$

(2) $$Y=DWC\left(\left(MLP\left(T_{K}\right)\right)\right)$$

(3) $$Y_{shift\;}=Shift_{G}\left(Y\right);T_{G}=Tokenize\left(Y_{shift}\right)$$

(4) $$\;\;\;\;Y=LN\left(F+MLP\left(GELU\left(T_{G}\right)\right)\right)$$

where G is for height, K is for width, DWC is for deep convolution, $Shift_{K}$ stands for Shift operation in width direction, $Shift_{G}$ stands for shift operation in the height direction, Tok stands for feature mapping operation, F stands for tokens gained by feature mapping operation, DWC stands for deep convolution, and LN for layer normalization. As shown in the above formula, firstly, the feature information obtained by convolution is obtained by shift operation and feature mapping operation along the width direction to obtain $T_{K}$, and then the output *Y* is obtained by *MLP* and *DWConv* operations on $T_{K}$. We carry out a displacement operation and a feature mapping operation along the height direction for the obtained output *Y* to get $T_{G}$. We carry out an MLP and GELU operations on $T_{G}$. After adding the output obtained with $T_{K}$, we carry out an *LN* operation. Our network enhances the recognition of local features of skin lesions by using TokMLP blocks to strengthen the association of elements with surrounding elements.

### 3.3. CTrans Module

**Figure 4:**
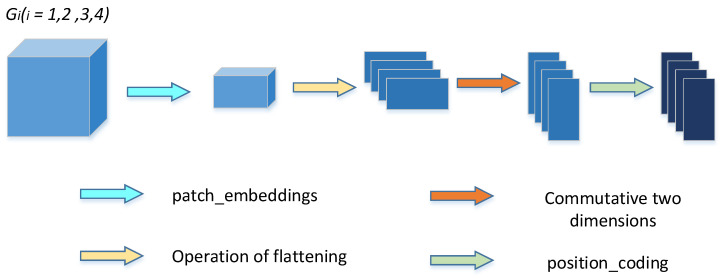
Multiscale feature embedding process.

The CTrans module we used is composed of two modules, CCT and CCA. CCT is a channel cross-fusion transformer, which is composed of three parts: multiscale feature embedding, multihead channel cross-attention, and multilayer perceptrons (*MLP*) (see Figure 4). It has the effect of fusing features from multiscale encoders. CCA is channel cross-attention, which has the effect of fusing features from multiscale encoders.

Firstly, the feature graph ($G_{i}\in R^{\frac{H\times W}{2^{j}}\times C_{i}}\left(i=1,2,3,4\;j=0,1,2,3\right)$) of its four skip connection layers (including those operated by the TokMLP block) is reconstructed by a multiscale feature embedding operation.

We first embed the feature graph $G_{1}\in R^{64\times 224\times 224}$ (as shown in Figure 4) and $G_{1}$ and then perform the patch_embeddings operation (that is, after a convolution operation with kernel size of (16, 16) and step size of 16). The output $P_{1}\in R^{64\times 14\times 14}$ is obtained, and then the output $F_{1}\in R^{64\times 196}$ is flattened. We perform a dimension swap operation on its length and width to obtain the output $J_{1}\in R^{196\times 64}$. Finally, we perform position coding operations on it to obtain the output $X_{1}\in R^{196\times 64}$. Similarly, for $G_{2}\in R^{128\times 112\times 112}$, $G_{3}\in R^{256\times 56\times 56}$, and $G_{4}\in R^{512\times 28\times 28}$ and after the operation to obtain new features ($X_{2}\in R^{196\times 128},\;X_{3}\in R^{196\times 256},\mathrm{and}\;X_{4}\in R^{196\times 512}$) in the same way. The four resulting results are entered into the encoder in sequence and then spliced according to their channel dimensions. Then, the output $X_{c}\in R^{196\times 960}$ obtained after concatenation.

**Figure 5:**
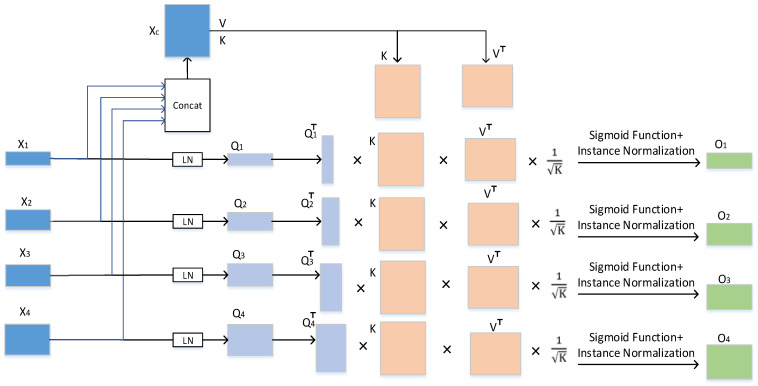
Operation schematic diagram of the transformer.

We first input $X_{i}$ and $X_{c}$ into the CCT module for transformer operation, and its working principle is shown in Figure 5.

(5) $$Q_{i}=X_{i}M_{d},K=X_{c}M_{K},\;\;V=X_{c}M_{V}$$

In Formula (5), $M_{d}\in R^{L_{i}\times b}$, $M_{K}\in R^{L_{C}\times b}$, and $M_{V}\in R^{L_{C}\times b}$ represent the weight of each input, respectively, $Q_{i}\in R^{L_{i}\times b}$, $K\in R^{L_{c}\times b}$, and $V\in R^{L_{c}\times b}$. *b* represents the sequence length (i.e., number of patches) and $L_{i}$ (i = 1, 2, 3, 4) represents the channel size of each jump connection layer. The channel sizes of the four jump connection layers in our experiment were 64, 128, 256, and 512, respectively.

The matrix $Z_{i}$ is generated using the cross-concern (CA) mechanism in the multihead cross-self-attention. We use the matrix $Z_{i}$ to weight the value *V*:

$$O_{i}=Z_{i}V^{⊤}=\sigma \left[\psi \left(\frac{Q_{i}^{⊤}K}{\sqrt{L_{C}}}\right)\right]V^{⊤}$$

(6) $$=\sigma \left[\psi \left(\frac{M_{Q_{i}}^{⊤}X_{i}^{⊤}X_{C}M_{K}}{\sqrt{L_{C}}}\right)\right]M_{V}^{⊤}X_{C}^{⊤}$$

where ψ represents the instance normalization operation, sigma is the softmax function.

The $O_{i}$ is computed for each $Q_{i}\;\left(i=1,\;2,\;3,\;4\right)$, and four $O_{i}$ are generated for each of the four inputs. In the case of “*S* many heads of attention”, its output can be calculated as follows:

(7) $$NC_{i}=\left(O_{i}{}^{1}+O_{i}{}^{2}+,\cdots ,+O_{i}{}^{S}\right)/S$$

(8) $$K_{i}=NC_{i}+MLP\left(LN\left(Q_{i}+NC_{i}\right)\right)$$

In Formula (7), $NC_{i}$ represents the output of multiheaded cross-self-attention. In Formula (8), *MLP* represents the multilayer perceptron in the CCT module, LN represents the layer normalization, and $K_{i}\left(i=1,\;2,\;3,\;4\right)$ represents the four outputs operated by the CCT module.

**Figure 6:**
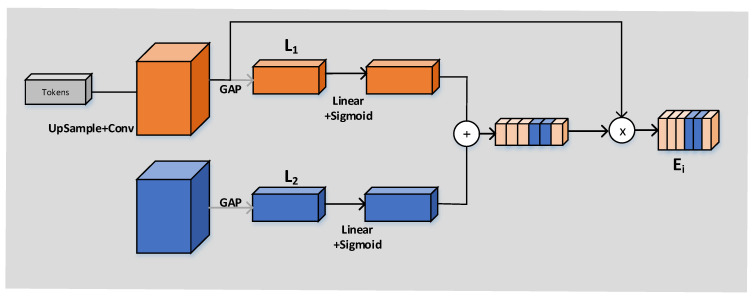
Channel cross-attention module.

The working principle of the CCA module is shown in Figure 6. The input of the CCA module is, respectively, every output $K_{i}\in R^{H\times W\times C}\left(i=1,\;2,\;3,\;4\right)$ obtained through the operation of the CCT module and every feature figure $N_{i}\in R^{H\times W\times C}\left(i=1,\;2,\;3,\;4\right)$ in the decoder. The vector $L_{x}\in R^{1\times 1\times C}\left(L=1,\;2\right)$ can be obtained by compressing the GAP layer’s space. The full connection layer should not only expand the convolutional layer into vectors but also classify each feature map. The idea of GAP is to combine the above two processes into one and carry them out together. The purpose of GAP we use is to solve the overfitting risk caused by fewer parameters of the full connection but also to achieve the same conversion function of the full connection. Then, linear and sigmoid operations were carried out, respectively, and finally (as shown in Figure 3), pixel point addition and pixel point multiplication were successfully carried out.

We obtain four outputs after CCA module operation $E_{i}$ ($E_{i}\in R^{H\times W\times C}\left(i=1,\;2,\;3,\;4\right)$), and we need to concatenate these four outputs, respectively, with the output $N_{i}$ ($N_{i}\in R^{H\times W\times C}\left(i=1,\;2,\;3,\;4\right)$) of the decoder obtained by the up-sampling operation in accordance with the channel dimension. After a convolution (kernel 1) and sigmoid operation on the final output, we obtain the final segmentation result graph. 

## 4. Experiments

In this section, we first introduce the datasets used in our experiments. Then, the evaluation metrics for the experiments are introduced. Next, we describe the parameter settings of the training process in detail. Finally, the loss function is introduced.

### 4.1. Datasets

**Figure 7:**
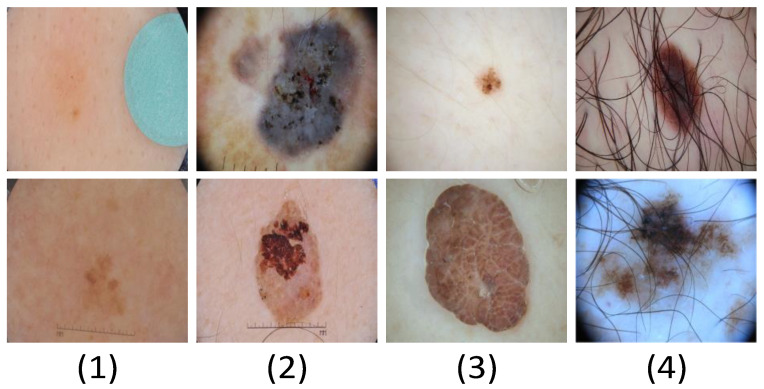
Challenges encountered in dermatological segmentation.

We used three accepted datasets for skin lesions (ISBI2016 [34], ISBI2017 [35], and ISBI2018 [36]) to validate the proposed network. The training set, verification set, and test set of the dataset are composed as shown in Table 1. The main types of skin injuries in the dataset are shown in Table 1 (as shown in Figure 7).

(1) There are small differences between normal skin and lesion images in the sample, which adds some challenges to the work of lesion image segmentation.
(2) There are obvious multilevel features inside and outside the lesion in the sample, which may cause the network to mislocate the lesion boundary.
(3) The size and shape of the lesions are basically irregular, and the boundaries are also blurred, making them difficult to identify.
(4) The image in the sample has interference factors such as hair, air bubbles, and other occluders, which will affect the segmentation accuracy of the network.

The training dataset of the ISIC2018 dataset provides 2594 labeled images of lesions. In addition, the validation set of this dataset consists of 100 unlabeled lesion images, and the test data consists of 1000 unlabeled lesion images. We need to redivide the training data of the original dataset into a training dataset, a verification dataset, and a test dataset, and the distribution ratio is 7:1:2.

The ISBI2017 dataset for training includes 2000 dermoscopic images of different resolutions with segmentation labels. Its test data consists of 600 dermoscopic images with corresponding segmentation markers. Regarding the distribution of this dataset, we used the training data of the original dataset as the experimental training dataset. At the same time, we divided the test data of the original dataset into a validation dataset and a test dataset, and the allocation ratio was 1:4.

The ISBI2016 dataset contains 900 training images in JPEG format and corresponding segmentation label images in PNG format, including 379 test images. For this dataset, we need to redistribute the original data. The scheme was as follows: we used the 900 training images provided by ISBI 2016 as the training set, and we used the test images of the original data as both validation and test datasets.

### 4.2. Evaluation Metrics

In this experiment, we used the dice coefficient (*Dice*) and intersection ratio (*IoU*) to evaluate the segmentation performance of the network. The evaluation index we used was calculated from four values: *TP*, *FP*, *TN*, and *FN*. When the predicted results are consistent with the actual results and both are positive, *TP* indicates that the prediction of the network is correct. When the predicted result is positive but its actual value is negative, it means that the model has made a wrong judgment, and *FP* means that the result predicted by the model is wrong. When the predicted result is consistent with the actual value and the predicted result is a negative value, *TN* indicates that the predicted value of the model is correct at this time. In the case that the predicted result is negative and its true value is positive, it indicates that the judgment result of the model is incorrect, and *FN* indicates that the predicted value of the model is wrong at this time. The calculation principle of the *Dice* and *IoU* indicators we use is shown in the following formula:

(9) $$Dice=\frac{2TP}{2TP+FN+FP}$$

(10) $$IoU=\frac{TP}{TP+FN+FP}$$

### 4.3. Experimental Setup

In our experiments, we did not use any of the network’s pretrained weights. The resolutions of images in the skin lesion dataset are different. In the experiment, we uniformly readjusted the resolution of lesion images to 224 × 224 for testing. The experiment ran on the Python 3.6 and Torch 1.8.1 platforms. We ran the experiments on two Tesla T4 GPUs (15 GB of memory), and then used the ADAM optimizer to optimize the results. Next, we set the learning rate, batch size, and epoch to 0.001, 24, and 200, respectively. The format of the dataset we used is a picture, and we used online data augmentation methods such as horizontal (vertical) flipping and random rotation for the data.

### 4.4. Loss Function

In addition to the discussion of the experimental setup, we also describe the loss functions used in the experiments. In our medical image segmentation model, we used binary cross-entropy loss (*BCE*) and dice loss (*Dice*) to train the network. It is stated as follows:

(11) $$L_{BCE}=-\frac{1}{W\times H}\overset{W\times H}{\underset{i=1}{\sum }}\left(y_{i}logx_{i}+\left(1-y_{i}\right)\log\left(1-x_{i}\right)\right)$$

(12) $$L_{Dice}=1-\frac{2TP}{\left(FN+TP\right)+\left(FP+TP\right)}$$

where $y_{i}$ represents the ground truth pixel.

## 5. Results

In this section, we perform ablation experiments on connections at four different locations in the CTrans module. Finally, we present the experimental results of the HMT network.

### 5.1. Ablation Experiment

**Figure 8:**
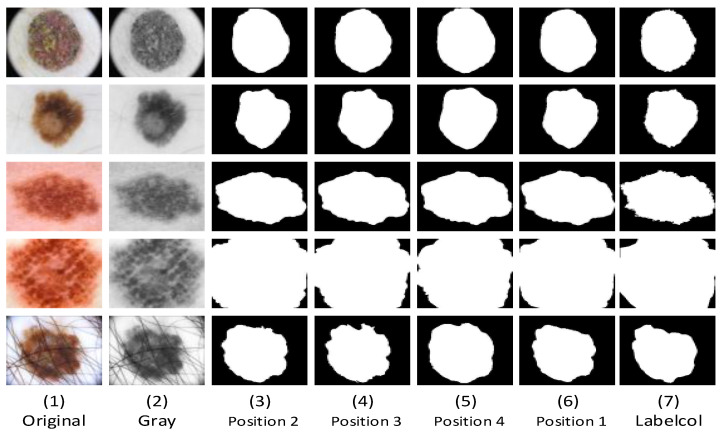
A visual comparison of each network in the ablation experiment. (**1**,**2**) The original image and grayscale image that were fed into the network. (**3**–**5**) The CTrans module is connected with the TokMLP block at the second, third, and fourth positions. Their segmentation effect is slightly worse, and their segmentation prediction graph is slightly greater than the labelcol graph. Compared with Figure 6, the segmentation performance is not ideal, and Figure 6 is closer to the label-column diagram. By comparing (**3**,**7**), Figure 6 is closer to the label diagram. By comparison (**3**–**7**), we can clearly see that the segmentation prediction graph is slowly approaching the label graph. By comparison, we can find that the position-one connection method can not only overcome the shortcoming of the CNN’s difficulty in capturing context connection but also achieve accurate segmentation of focal local features.

In order to better demonstrate the effect of the experimental method mentioned in this paper. We conducted related ablation experiments on the ISIC2018 dataset. In our ablation experiment, as shown in the connection position of the CTrans module in Figure 1, the sequence numbers of its connection positions are 1, 2, 3, and 4 from bottom to top. We tested the segmentation effect of concatenating TokMLP blocks at different locations on the dataset. We compared the effects of different connection methods between the two modules on the effect of lesion segmentation. As shown in Figure 8, we can observe that there are obvious differences in the segmentation results due to the different connection methods of the two modules. From the figure, it can be clearly seen that when the TokMLP block is connected to the first position of the CTrans module, it can obtain clearer predicted segmentation maps, especially when lesions have different locations and shapes.

In addition, we also made relevant statistics and comparisons on the DC and IOU values of each dataset. As shown in Table 2, Table 3 and Table 4, it is clear from the data in the table that the segmentation index is increasing from position 4 to position 1. It can be seen that our network can not only realize the learning of the association between remote contexts but also realize the understanding of the local features of the lesions to realize the accurate segmentation of the skin lesions. In addition, we also conduct relevant statistics on the three datasets (ISBI2016, ISBI2017, and ISIC2018) through the evaluation indicators (IOU and DC) (Table 2, Table 3 and Table 4). It can be confirmed by statistical data that the TokMLP block is connected to the first position. The segmentation results are more feasible than the other three schemes.

### 5.2. Comparative Experiment

**Figure 9:**
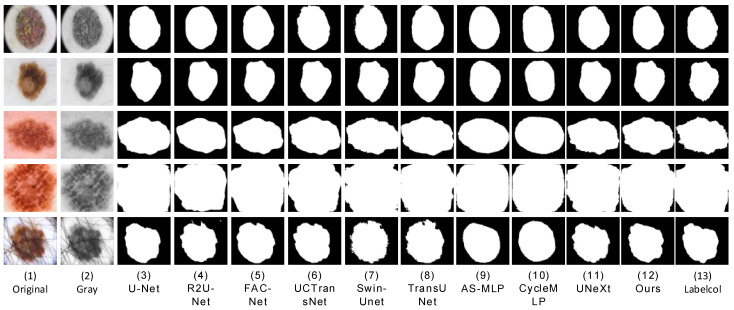
On ISIC2018, a visual segmentation comparison map of each compared network is displayed. (**1**,**2**) The input original image and grayscale image, respectively, and (**13**) the label-col map. (**3**–**11**) The comparison network segmentation results, and (**12**) the method in this paper segmentation results. It can be seen that the segmentation results of the method in this paper are the closest to the label-column diagram.

**Figure 10:**
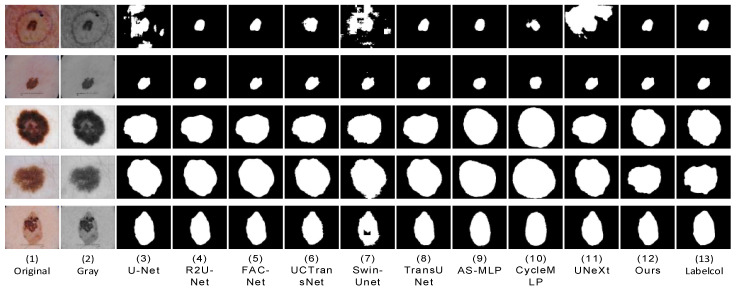
At ISBI2017, a visual segmentation comparison map was created for each compared network. (**1**,**2**) The input original image and grayscale image, and (**13**) the label-col map. (**3**–**11**) The segmentation results of the comparison networks, respectively. (**12**) The segmentation result of our proposed method from which we can see that the segmentation result of our proposed algorithm is closest to the label-column map.

**Figure 11:**
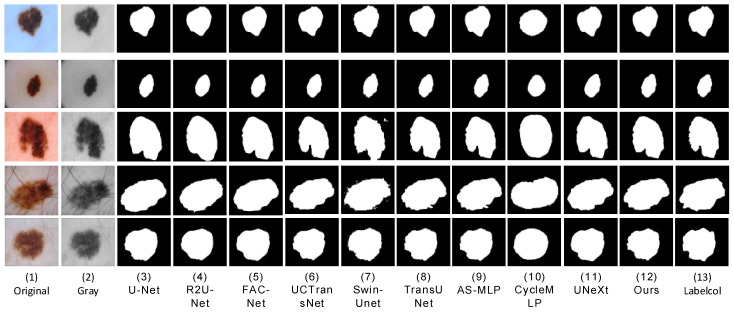
At ISBI2016, a visual segmentation comparison map was created for each compared network. (**1**,**2**) The input original image and grayscale image, and (**13**) the label-col map. (**3**–**11**) The segmentation results of the comparison networks, respectively. (**12**) The segmentation result of our proposed method from which we can see that the segmentation result of our proposed algorithm is closest to the label-column map.

We conducted comparative experiments on proposed segmentation networks and mainstream segmentation networks, including UNet [7], R2U-Net [8], FAC-Net [9], UC TransNet [26], TransUNet [27], Swin-Unet [28], UNeXt [14], AS-MLP [29], and CycleMLP [30]. In order to ensure the fairness of the experimental comparison, we conducted experiments in the same computing environment with the most suitable parameters for each network. We apply each network to three datasets (ISBI2016 [33], ISBI2017 [34], and ISIC2018 [35]). The prediction result images of skin lesions obtained through network training segmentation are shown in the following three figures (Figure 9, Figure 10 and Figure 11). We can see that U-Net [7] was frequently unable to accurately segment the difficult complex lesion boundaries. The segmentation effect of R2U-Net [8] and U-Net [7] in the popular CNNs was worse than that of FAC-Net [9]. However, it can be seen from the figure that CNNs represented by FAC-net often lost important foreground information when discarding useless background information. The transformer based approach can effectively remedy this problem with an understanding of the connections between remote contexts. At present, the mainstream UCTransNet [26], TransUNet [27], based on the transformer architecture, and UNeXt [14], based on the MLP architecture, both achieve relatively high segmentation accuracy. Its performance may be better than or equal to that of the latest CNNs architecture network based on FAC-Net [9]. In addition, it can be seen from the figure that UNeXt based on MLP method can achieve accurate location of focal local features through axial displacement operation. However, in the first line of Figure 5, we can clearly see that U-Net [7], Swin-Unet [28], and UNeXt [14] all have unclear segmentation errors. This is due to the interference factors of the marks used to mark the lesion outside the lesion site in the original image, which makes these networks unable to accurately identify the characteristic information of the lesion, and our method can still maintain accurate segmentation performance. The segmentation effect of AS-MLP [29], CycleMLP [30], and other networks was slightly worse than that of FAC-Net [9]. The segmentation diagram clearly shows that while the segmentation effect of FAC-Net [9] was better, the problem of not detailed segmentation of focal boundaries persists in FAC-Net [9] when compared to the results of HMT-Net segmentation proposed by us. In the comparison of the result figures, it can be clearly seen that the segmentation effect of the hybrid network HMT-Net proposed by us was significantly better than that of the currently commonly used CNNs segmentation network. Not only that, but it was also better than the more popular transformer and MLP split networks.

In addition to intuitive comparisons, we also performed statistical comparisons of the experimentally obtained data (IOU and DC) on three datasets (ISBI2016, ISBI2017, and ISIC2018). As can be seen from the following three tables (Table 5, Table 6 and Table 7), the method we used was superior to the current relatively new transformer-based and MLP-based networks in these two indicators. Moreover, compared with the state-of-the-art convolutional neural network, FAC-Net, the method used in this paper shows significant improvements on all three datasets. By comparing the IOU and DC index values on three datasets (ISBI2018, ISBI2017, and ISBI2016), the module we used was more robust in improving the results of skin lesion segmentation.

## 6. Conclusions

Accurate and efficient segmentation of medical images is a key step in clinical skin lesion analysis, and diagnosis. In this work, we designed a hybrid encoding network model, HMT-Net, from the perspective of channel encoders, with the aim of providing an accurate and reliable automatic segmentation algorithm for skin lesion images. By using the CTrans module based on the transformer architecture, we effectively improved the network’s computing power for the global information correlation of lesions. Thus, the ability of the network to identify the global foreground information of lesions was improved. At the same time, we use a marked MLP block with axial displacement, through different axial displacement operations, to enhance the correlation between pixels and adjacent elements, thereby improving the network’s attention to local feature information. It can effectively solve the problem of weak boundary information recognition existing in lesion image segmentation. Through in-depth analysis and experimental demonstration, we demonstrate the advantages of our HMT-Net model. It can indeed learn the global information of the image more effectively, and we can improve the network’s understanding of local information by enhancing the connection between adjacent pixels. We further combine the two ideas here to achieve precise and effective segmentation of skin lesions. Our future work will be carried out from the two aspects of lightweight network and improving the understanding of network local information.

## Figures and Tables

**Table 1:** The distribution of the dataset.

Dataset	Train	Verify	Test
ISIC2018	1815	259	520
ISBI2017	2000	150	600
ISBI2016	900	379	379

**Table 2:** Ablation experiments on the ISIC2018 dataset.

Method	Dice (%)	IoU (%)
Position 1	82.39	89.35
Position 2	82.18	89.15
Position 3	82.26	89.18
Position 4	81.99	89.15

**Table 3:** Ablation experiments on the ISBI2017 dataset.

Method	Dice (%)	IoU (%)
Position 1	75.53	84.93
Position 2	74.83	83.40
Position 3	74.23	84.86
Position 4	74.54	83.24

**Table 4:** Ablation experiments on the ISBI2016 dataset.

Method	Dice (%)	IoU (%)
Position 1	83.98	91.33
Position 2	83.78	91.23
Position 3	83.64	91.00
Position 4	83.75	91.07

**Table 5:** ISIC2018 evaluation indicators of each comparison network.

Method	Dice (%)	IoU (%)
U-Net * (2015)	77.43	87.13
R2U-Net * (2018)	78.85	88.05
FAC-Net * (2021)	80.4	88.9
TransUNet * (2021)	80.04	87.7
Swin-Unet * (2021)	80.02	87.73
UCTransNet * (2022)	80.15	88.13
AS-MLP * (2021)	78.21	86.81
CycleMLP * (2021)	71.4	81.88
UNeXt * (2022)	78.22	86.32
Ours	82.39	89.35

* represents the corresponding relationship between the split network and its birth year.

**Table 6:** ISBI2017 evaluation indicators of each comparison network.

Method	Dice (%)	IoU (%)
U-Net * (2015)	68.3	80.7
R2U-Net * (2018)	67.8	80.37
FAC-Net * (2021)	73.85	82.57
TransUNet * (2021)	72.26	81.37
Swin-Unet * (2021)	67.5	78
UCTransNet * (2022)	69.2	78.8
AS-MLP * (2021)	71.6	81.21
CycleMLP * (2021)	65.22	76.19
UNeXt * (2022)	71.7	81.1
Ours	75.53	84.93

* represents the corresponding relationship between the split network and its birth year.

**Table 7:** ISBI2016 each comparison network evaluation indicator.

Method	Dice (%)	IoU (%)
U-Net * (2015)	81.51	88.63
R2U-Net * (2018)	80.59	89.38
FAC-Net * (2021)	82.38	90.2
TransUNet * (2021)	81.93	89.12
Swin-Unet * (2021)	76.69	65.29
UCTransNet * (2022)	81.95	89.22
AS-MLP * (2021)	79.94	88
CycleMLP * (2021)	72.92	83
UNeXt * (2022)	81.61	88.92
Ours	83.98	91.33

* represents the corresponding relationship between the split network and its birth year.

## Data Availability

We used two classical dermoscopy datasets to evaluate the proposed segmentation network. They are the ISIC2018 dataset, ISBI2017 dataset, and ISBI2016 dataset. The URL of these datasets is https://challenge.isic-archive.com/data (accessed on 27 July 2021).
